# Determination of Polyvinyl Acetate in Chewing Gum Using High-Performance Liquid Chromatography–Evaporative Light Scattering Detector and Pyrolyzer–Gas Chromatography–Mass Spectrometry

**DOI:** 10.3390/foods9101473

**Published:** 2020-10-15

**Authors:** Sol Sim, Young-Min Kim, Yeong-Ju Park, Mohammed Xain Siddiqui, Yejin Gang, Jihyun Lee, Chan Lee, Hee-Jae Suh

**Affiliations:** 1 Department of Food Science, Sun Moon University, Asan, Chungchengnam-do 31460, Korea; s_soli@naver.com (S.S.); qkr9578@naver.com (Y.-J.P.); 2 Department of Environmental Engineering, Daegu University, Gyeongsan 38543, Korea; ymk@daegu.ac.kr; 3 Department of Environmental Sciences and Biotechnology, Hallym University, Chuncheon 24252, Korea; mohammedxainsiddiqui@gmail.com (M.X.S.); riveryj1215@gmail.com (Y.G.); 4 Advanced Food Safety Research Group, BrainKorea21 Plus, Department of Food Science and Technology, Chung-Ang University, 4726, Seodong-daero, Anseong, Gyeonggi-do 17546, Korea; jihlee@cau.ac.kr

**Keywords:** polyvinyl acetate, glazing agent, high-performance liquid chromatography with an evaporative light scattering detector (HPLC–ELSD), pyrolyzer–gas chromatography–mass spectrometry (Py–GC–MS)

## Abstract

Polyvinyl acetate (PVAc) is used in various adhesive, paint, and transparent tape applications. It is also used as a food additive in food manufacturing to make chewing gum and fruit and vegetable glazes; however, guidelines on the amount of food additives that is used have not yet been established. In this study, a method was developed for analysis of polyvinyl acetate (PVAc) using high-performance liquid chromatography with an evaporative light scattering detector (HPLC–ELSD) and pyrolyzer–gas chromatography–mass spectrometry (Py–GC–MS). The analytical methods were applied to commercially available chewing gum. In the HPLC–ELSD analysis, the linearity was acceptable (R^2^ > 0.999), and the limits of detection and quantification were 22.2 and 67.3 µg/mL, respectively. The accuracies of PVAc were 87–115% at spike levels of 200–1000 µg/mL for the intra- and inter-day tests. The contents of PVAc in the chewing gum samples were n.d. (not detected)—13.8 g/kg. The presence of PVAc in chewing gum was verified with Py–GC–MS analysis, finding the typical pyrolysates of PVAc, such as acetic acid, benzene, toluene, styrene, indane, naphthalene, and acenaphthene. The developed analytical methods can be applied for successful identification of PVAc in chewing gum.

## 1. Introduction

**Figure 1:**
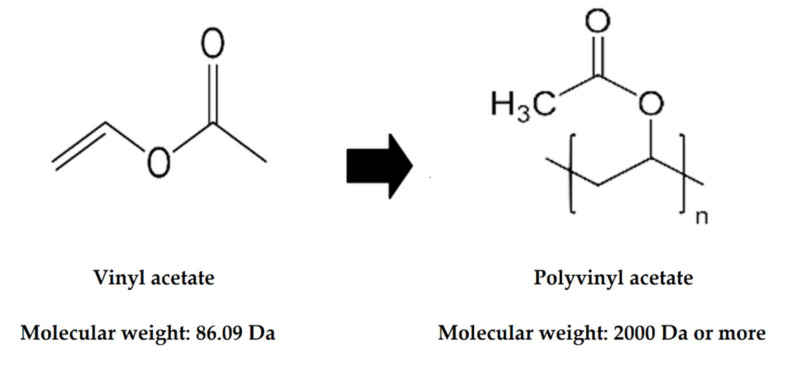
Structures of vinyl acetate and polyvinyl acetate.

Polyvinyl acetate (PVAc) is a polymer of vinyl acetate that is obtained by reacting acetic acid with ethylene, which is produced from petroleum (Figure 1). It is a colorless or pale-yellow glassy mass that is insoluble in water and oils but completely hydrolyzed in esters and alcohols such as ethyl acetate and ethanol. PVAc is used in various adhesive, paint, printing ink, and transparent tape applications. It is also used as a food additive in food manufacturing to make gum, fruit and vegetable glazes, and gum bases [1,2]. It has been allowed as a food additive in Korea since June 1962, and has been used as a chewing gum base or as a coating agent for chewing gum or fruits and vegetables; however, guidelines for the amount that is used have not yet been established [3]. PVAc is allowed to be used as a vegetable coating agent and chewing gum base in Japan, and as a chewing gum base in the US, and guidelines for its use in both countries are set according to good manufacturing practices, as in Korea [4,5].

According to Korean statistics on the domestic production of foods and food additives and imported food products, the quantity of PVAc used has been decreasing in both imports and domestic products since 2014; however, production statistics show it has been used as a food additive continuously up to 2019 [3]. Because there is no official verified analytical method for PVAc in Korea, it is difficult to figure out the amounts that are being used in food products. Furthermore, no official accredited analytical method has been established in any other country.

Food additives are frequently consumed by humans in their diets, so it is critical to understand their toxicities and safe usage levels. It has been shown that PVAc can cause symptoms like irritation of the eyes, skin, and digestive tract at a dose of 25 g/kg body weight in rats and mice [6]. Carcinogen research by the International Agency for Research on Cancer has shown that PVAc is in Group 3 and not carcinogenic to humans, but its main component, vinyl acetate, is carcinogenic. Because of the designation of vinyl acetate as a Group 2B carcinogen, it is important to determine the exact amount of PVAc used as a food additive to ensure the degree of polymerization allows for safe use [7].

Because PVAc has a molecular weight of 2000 Da or more (Figure 1), qualitative analysis can be performed by identifying the degradation products after decomposition into low molecular weight materials [8]. In the pyrolysis of PVAc, acetic acid is released when the CO bond between the side chain of the polyvinyl acetate and the main backbone is broken. At temperatures of around 400 °C, acetic acid and aromatic hydrocarbons are produced by decomposition of the polymer backbone and cyclization at the olefin end [9,10]. Renuka Devi and Madivanane (2012) recorded Fourier transform infrared (FTIR) spectra of PVAc that showed characteristic absorptions, chemical bonding, chemical functions, and group properties depending on the spectral range, but it was very difficult to calculate the amount of PVAc used from these results [11]. To date, some qualitative analyses of PVAc have been performed in the field of polymer chemistry, but few have reported qualitative or quantitative analyses of PVAc in food.

**Figure 2:**
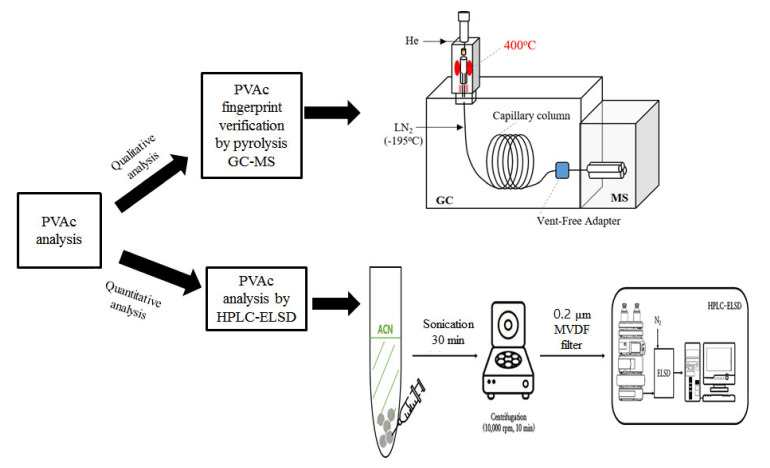
Strategy of polyvinyl acetate (PVAc) analysis and its analytical conditions. GC-MS, gas chromatography–mass spectrometry; HPLC–ELSD, high-performance liquid chromatography with an evaporative light scattering detector; LN_2_, liquid nitrogen gas; ACN, acetonitrile; PVDF, polyvinylidene fluoride.

In this study, qualitative verification of PVAc was performed using a pyrolyzer–gas chromatography–mass spectrometry (Py–GC–MS). To confirm that PVAc was detected, qualitative analysis was performed using positive containing PVAc and negative samples without PVAc. The evaporative light scattering detector (HPLC–ELSD) analysis conditions and pretreatment methods for PVAc analysis were optimized according to existing methods for other polymer resins that have similar physical and chemical properties and structures to PVAc (Figure 2). Validation of the developed method was performed using the linearity, limit of detection (LOD), limit of quantification (LOQ), precision, accuracy, and cross-laboratory verification.

## 2. Materials and Methods

### 2.1. Reagents and Reference Standard

The standard of PVAc (USP Reference Standard) was purchased from Sigma-Aldrich (Darmstadt, Germany). HPLC-grade water, methanol, and acetonitrile were obtained from Daejung Chemicals and Metals (Gyeonggi-do, Korea). A 0.2-μm polyvinylidene fluoride (PVDF) filter was obtained from Whatman (Maidstone, England).

Stock standard solution of PVAc was prepared in acetonitrile at 1000 mg/L and stored at −4 °C until use. Dilution of standard was used to prepare concentrations of 50, 100, 200, 300, 400, 500, 600, 700, 800, and 900 mg/L. All dilution standards were used immediately after preparation.

### 2.2. Py–GC/MS Analysis

A micro-furnace Py–GC–MS (7890A/5975 Inert, Agilent Technologies, Santa Clara, CA, USA) (Figure 2) was used to determine the presence of PVAc in chewing gum. A PVAc reference standard (RS) and commercial chewing gum (CG) were used as the standard polymer and actual sample, respectively.

For the Py–GC–MS analysis, 0.3 ± 0.05 mg of the RS or CG was placed in a deactivated metal cup and inserted into the pyrolyzer furnace, which was preheated to 400 °C. The pyrolysis product vapor emitted from the furnace was transferred to a metal capillary column (UA-5, 30 m × 0.25 mm i.d. × 0.25 µm film thickness) via a split/splitless inlet (320 °C, split ratio 200:1) and cryofocused at the front of the column using liquid nitrogen (−195 °C) for 3 min. After cryofocusing, the pyrolysis products were separated on the column under non-isothermal GC oven heating, detected using a quadrupole MS, and identified by comparing the mass spectra with those in the NIST 08 (Agilent Technologies, Santa Clara, CA, USA) and F-Search (Frontier Laboratories, Koriyama, Japan) libraries. The Py–GC–MS operation conditions applied in this study are given in Table S1.

### 2.3. HPLC–ELSD Analysis

Quantitative analysis of PVAc by HPLC–ELSD was performed using a 1260 Infinity system (Agilent Technologies, Santa Clara, CA, USA) coupled to an evaporative light scattering detector (Agilent Technologies, Santa Clara, CA, USA). The analysis method was modified from that in a Waters Corporation application note for polyethylene glycol (PEG) (2013). A HyperClone ODS column (5 μm, 4.6 mm × 250 mm, GL Sciences, Shinjuku, Japan) was used at a temperature of 30 °C for chromatographic analysis; the mobile phase contained water and methanol and was used in gradient elution as follows: 0–6 min: 50% A:50% B; 6–8 min: 50% A to 40% A:50% B to 60% B; 8–10 min: 40% A to 25% A:60% B to 75% B; 10–12 min: 25% A to 10% A:75% B to 90% B; 12–14 min: 10% A to 0% A:90% B to 100% B; and 14–17 min: (re-equilibrium stage): 0% A to 50% A:100% B to 50% B. The flow rate of the elution was 1 mL/min and the evaporator and the nebulizer temperature for the ELSD were 50 and 70 °C, respectively.

To extract PVAc, 1 g of reference standard was placed in a centrifuge tube, and 5 mL of each of acetonitrile, ethanol, and methanol were added to the tube, followed by sonication at 40 °C and 500 W (UC-20, Lab Companion, Billerica, MA, USA). Preliminary results indicated that the PVAc did not dissolve in ethanol, methanol, or hexane, leaving a visible residue, but completely dissolved after sonication with acetonitrile for 30 min. If the solubility is similar, the extraction method using a mixed solvent may be used, as in the study of Bianco et al. (2013) [12]. However, since the solubility of PVAc was completely dissolved in acetonitrile without any residue among the four solvents, we chose acetonitrile as the PVAc extraction solvent. To extract the PVAc contained in the chewing gum, a sample of gum was precisely cut and weighed (1 g) into a centrifuge tube. After addition of 5 mL of acetonitrile to the tube and sonication for 30 min, the mixture was centrifuged for 10 min at 10,000 rpm, and the upper layer was removed and filtered through a membrane filter (pore size 0.2 µm) before HPLC analysis. All samples were analyzed in triplicate. To verify the method for PVAc extraction from commercially available chewing gum, it was purchased from local and internet markets, and recovery (accuracy) and precision tests were performed.

### 2.4. Method Validation for Quantitative Analysis

For verification of the quantitative HPLC method, the linearity, LOD, LOQ, accuracy, and precision were evaluated. Standard solutions of PVAc were prepared at a concentration range of 200–1000 µg/mL, as described in the Korean Ministry of Food and Drug Safety guidelines 145 [13]. A calibration curve was prepared from the peak areas obtained after analysis by HPLC, which was performed three times. The R^2^ values of the slope obtained from the calibration curve were calculated to confirm the linearity.

The LOD and LOQ for the PVAc were determined according to the ICH’s Harmonized Tripartite Guideline as follows [14,15]. After calculating the slope (S) of the calibration curve obtained at low concentrations (30, 50, and 100 µg/mL) and the standard deviation (σ) of the result obtained for seven repeat measurements of the intermediate analytical concentration (50 µg/mL), the LOD and LOQ were calculated as 3.3 and 10 σ/S, respectively. To assess the accuracy and precision, PVAc recoveries were obtained from blank gum samples (a gum that does not contain PVAc) spiked at several concentrations with PVAc. The accuracy and precision were expressed as the recoveries and relative standard deviations (%RSD), respectively, of the blank gum samples spiked at the nine different concentrations, namely, 200, 300, 400, 500, 600, 700, 800, 900, and 1000 µg/mL. The accuracy and precision were calculated from inter- and intra-day tests. For the inter-day test, blank gum samples were spiked at nine concentrations (as mentioned above) with PVAc and then measured once a day for 3 days. For the intra-day test, blank gum samples were spiked at the same concentrations as the intra-day test with PVAc and analyzed five times in 1 day. The accuracy was evaluated using recoveries calculated by comparing the PVAc concentrations obtained from the spiked blank gum samples with theoretical concentrations. The precision was evaluated using the repeatability, which was calculated as the %RSD between the results obtained from the recovery tests.

To ensure the validity of the quantitative method, blank gum samples spiked at three different concentrations (300, 500, and 700 µg/mL) were tested in three different laboratories and the results were cross-validated between the laboratories. The measurements included the linearity, recovery, and relative standard deviation (%RSD). For the calibration curve, measurements for the five concentrations (200, 400, 600, 800, and 1000 µg/mL) of PVAc were repeated three times in each laboratory. The R^2^ values of the slope obtained from the calibration curve were calculated to confirm the linearity. The spiked samples were analyzed in the three laboratories using the same pretreatment and HPLC methods proposed in this study, and the recoveries and cross-laboratory RSD (%) were calculated.

## 3. Results and Discussion

### 3.1. Py–GC–MS Analysis

**Figure 3:**
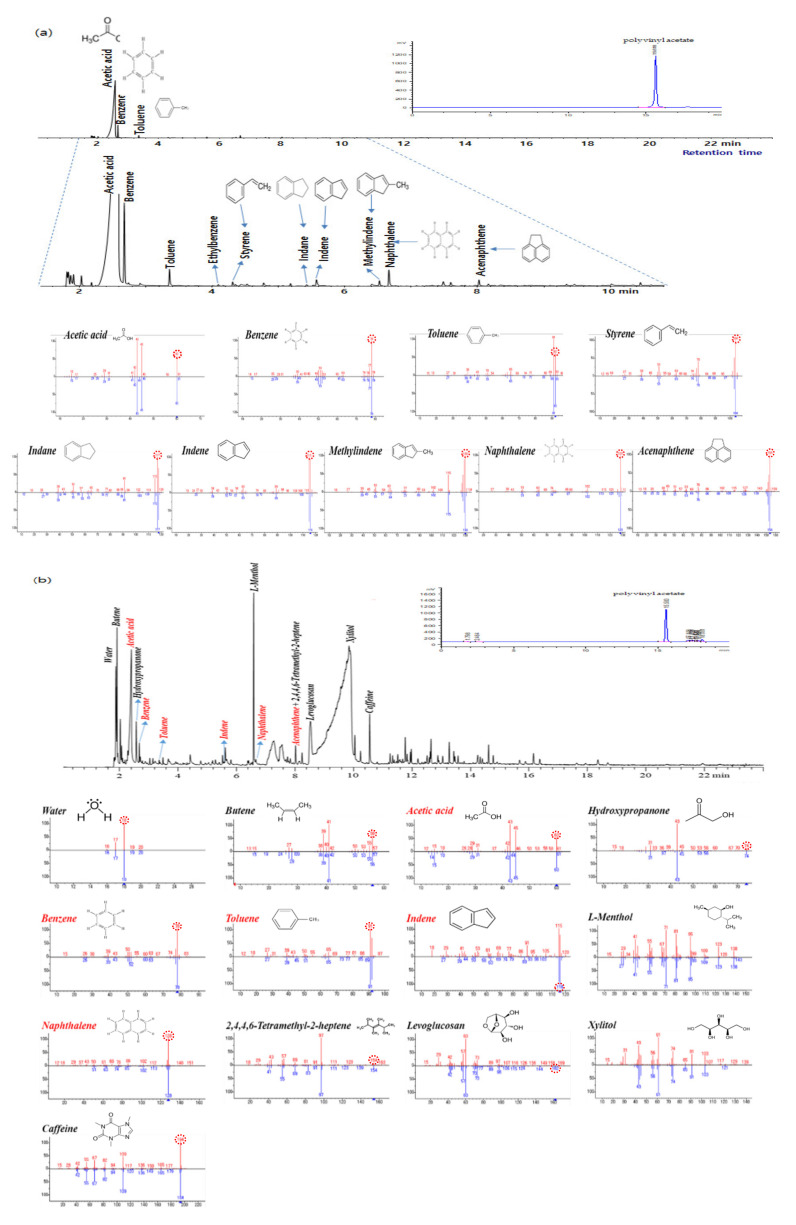

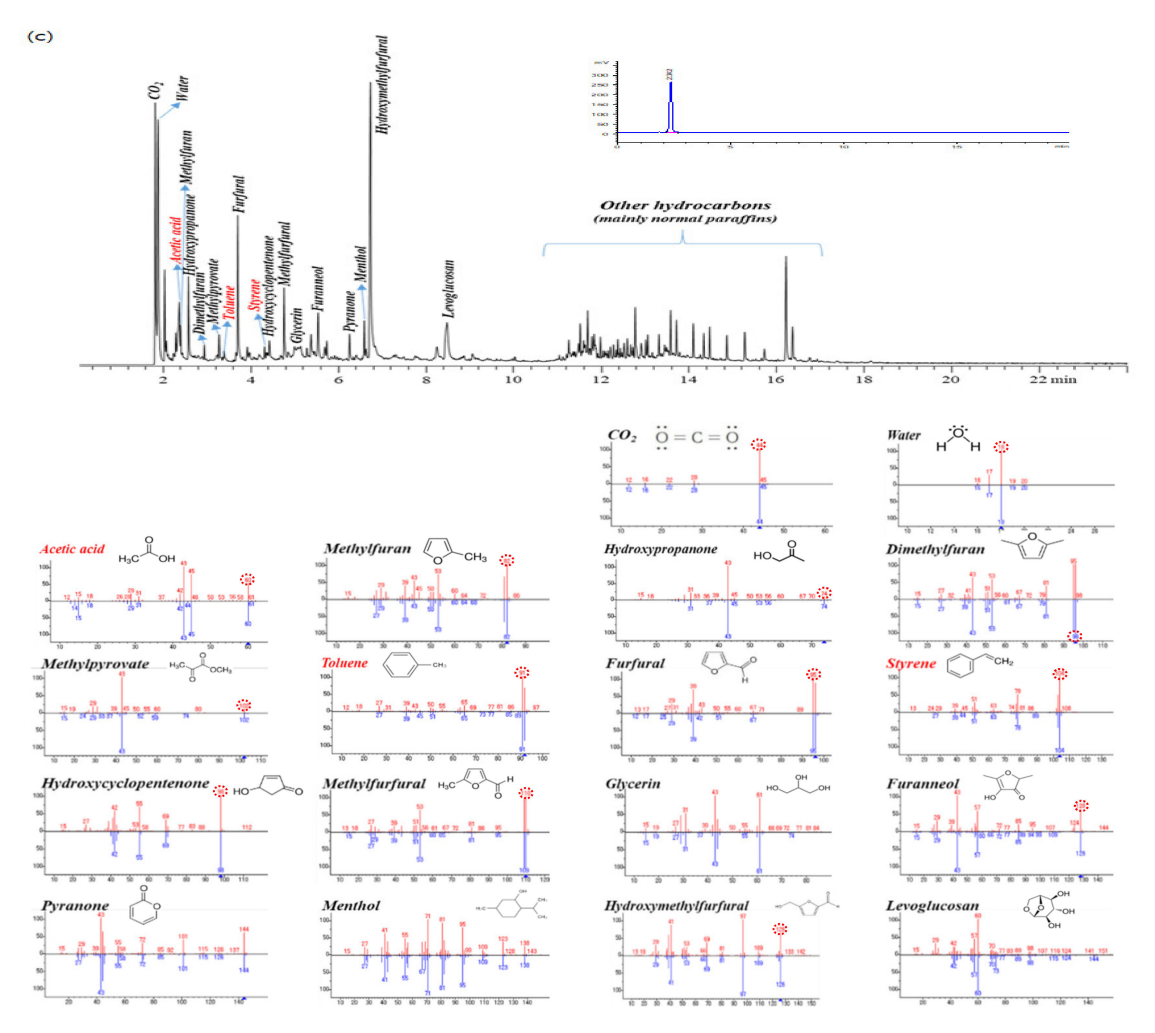
Total ion chromatograms of the PVAc reference standard (**a**), positive sample gum (**b**), and negative sample gum (no PVAc) (**c**) obtained from the Py–GC–MS analysis and a chromatogram of those samples from the HPLC–ELSD analysis.

Figure 3a shows the pyrogram obtained from the flash Py–-GC–MS analysis of RS at 400 °C. Typical pyrolysates of PVAc, such as acetic acid, benzene, toluene, indane, indene, methylindenes, naphthalene, and acenaphthene, which have been reported in the literature (Sellier et al.), were observed in the pyrogram [8]. The acetic acid peak was more intense than the peaks of the other pyrolysates of PVAc. During pyrolysis, acetic acid is emitted from decomposition of the C–O bonds between the side chain and main backbone of the PVAc in the low temperature region. Decomposition of the olefin backbone is enhanced in a second decomposition step in the high temperature region. As well as acetic acid, high temperature decomposition can produce aromatic hydrocarbons via decomposition of the polymer backbone and cyclization of the olefin intermediates. In the case of isothermal pyrolysis of PVAc, the yields of acetic acid and aromatic hydrocarbons change with the applied temperature. Uyar et al. found that the yield of acetic acid decreased and the yield of aromatic hydrocarbons increased when the pyrolysis temperature was increased from 360 °C to 440 °C, suggesting that secondary cracking of acetic acid and formation of aromatic hydrocarbons increased at higher temperatures [10]. Because other polymer components of chewing gum can also produce aromatic hydrocarbons (Tsuge et al.), we selected acetic acid as an indicator of the presence of PVAc in chewing gum to achieve the aim of this study [9]. To increase the intensity of the acetic acid peak without unnecessary decomposition of other polymers in the chewing gum matrix, we selected a pyrolysis temperature of 400 °C.

To verify the analytical method for PVAc developed in this study, the same equipment and methods were used to analyze a chewing gum sample, which does not contain PVAc, as a blank gum and chewing gum containing PVAc as a PVAC-positive gum. Figure 3b shows the pyrogram obtained from the Py–GC–MS analysis of the positive gum at 400 °C. Peaks for butane, hydroxyl propanone, menthol, levoglucosan, xylitol, and caffeine were monitored together with those of the typical pyrolysates of PVAc. Xylitol and caffeine are additives that are mixed together with the gum base polymer mixture to improve the functionality of chewing gum [16,17]. Levoglucosan is a typical pyrolysate of cellulose, which is one of the main base polymers in chewing gum [18]. Peaks for the typical pyrolysates of PVAc were observed in the pyrogram (Figure 3b) among the other monitored peaks, which showed that PVAc could be detected in chewing gum by this method. It is possible that quantification of PVAc in chewing gum could be achieved using the acetic acid peak; however, a more detailed study of this is required because the yield of acetic acid could change because of the interactions between acetic acid and other pyrolysis products during pyrolysis, which could lead to changes in the acetic acid peak area. For the blank gum, only small amounts of three components (acetic acid, toluene, and styrene) were detected (Figure 3c). In the blank gum, no indene, naphthalene, or acenaphthalene were detected. If the blank gum contained PVAc, the pyrogram would contain peaks for the same chemicals observed in the PVAc-positive gum; however, none of these chemicals were detected in the blank gum. Therefore, our results showed that the blank gum did not contain PVAc. A number of peaks were observed from 11 to 16 min in the blank gum pyrogram for hydrocarbons that were not observed in the PVAc-positive results, and these were believed to be derived from natural chicle.

### 3.2. HPLC–ELSD Analysis

As a first step in PVAc analysis, a preliminary experiment was performed using the vinyl acetate method proposed by the Joint Expert Committee on Food Additives (JECFA, 2015). This method, which uses HPLC with photodiode array detection (205 nm), can successfully detect vinyl acetate but is not suitable for PVAc determination because it is not separated [19]. However, because PVAc is a polymer of vinyl acetate, it can be detected as vinyl acetate after a depolymerization treatment. Unfortunately, depolymerization is time-consuming and expensive and it is very difficult to determine whether the peaks originate from vinyl acetate or depolymerized PVAc. Therefore, depolymerization of PVAc is not an appropriate method for analysis of chewing gum to detect PVAc.

In this study, a modification of an analytical method for PEG in its polymer form (Peter et al. (2013) was used for the PVAc analysis [20]. PVAc could be separated and detected using HPLC with evaporative light scattering detection (ELSD) and a reversed-phase column (Hyperclone™ ODS columns) with a gradient elution using water and methanol. A peak for the PVAc standard was detected at 15.6 min in the HPLC–ELSD chromatogram for a 25 min analytical run (Figure 3). In addition, PVAc was detected at the same time (15.6 min) in the chromatogram obtained for the commercial gum samples and analyzed under the same conditions.

### 3.3. Method Validation of Quantitative Analysis

#### 3.3.1. Linearity, LOD, and LOQ

To verify the HPLC method for quantitative analysis of PVAc, the linearity of the calibration curve was determined using the PVAc standards with a concentration range of 200–1000 µg/mL. The calibration curve showed excellent linearity (R^2^ > 0.999). In addition, the LOD and LOQ were calculated using statistical methods and were found to be 22.2 µg/mL and 67.3 µg/mL, respectively (Table 1) [14,15].

#### 3.3.2. Matrix Effect

The matrix effect was determined by comparing the slopes of the standard calibration curve and the matrix-based calibration curve obtained from the spiked blank sample (does not contain PVAc) by applying the method of the previous study [21]. For the matrix-based calibration curve, blank gum samples were spiked with PVAc at five concentrations (200, 400, 600, 800, and 1000 µg/mL) and the recoveries were determined using the method developed in this study. The R^2^ of the two calibration curves showed excellent linearity of >0.999, and there was no difference between the slopes of the two calibration curves. The results of the F- and t-tests between the two calibration curves also agreed. The recoveries for PVAc were calculated using both standard and matrix-based calibration curves. The recoveries obtained were very similar at 89.9–115.3% for the standard calibration curves and 87.6–113.3% for the matrix-based calibration curves. Therefore, all subsequent validation parameters were evaluated using the standard calibration curve.

#### 3.3.3. Accuracy and Precision

The accuracy was evaluated using recovery tests for blank gum spiked with PVAc at 200, 300, 400, 500, 600, 700, 800, 900, and 1000 µg/mL for the intra-day and inter-day tests. The recovery ranges in the intra- and inter-day tests were 98.1–115.0% and 86.6–115.3%, respectively, as shown in Table 1. These recoveries met the criterion of 70.0–120.0% recommended in the European Union (EU) Directive SANCO/2007/3131 (EC, 2007) [22].

The precision was evaluated in intra- and inter-day tests. The repeatability was calculated as the relative standard deviation of the intra-day analysis results obtained from the recovery tests repeated five times a day using a blank gum spiked with PVAc at 200, 300, 400, 500, 600, 700, 800, 900, and 1000 µg/mL. The repeatability ranged from 0.4–9.0% and was lower than 20%, which is the recommendation in the EU Directive SANCO/2007/3131 (EC, 2007) [22]. The intermediate precision was calculated as the relative standard deviation of results from the inter-day analysis on three separate days using samples spiked with PVAc at 200, 300, 400, 500, 600, 700, 800, 900, and 1000 µg/mL. The intermediate precision range was 0.6–7.5% and met the recommendation (RSD < 20%) in EU Directive SANCO/2007/3131 (EC, 2007) [22].

#### 3.3.4. Cross-Laboratory Test

To validate the HPLC method for PVAc, cross-laboratory tests were performed in three different laboratories on blank gum samples that were spiked at the same concentrations (300, 500, and 700 µg/mL). These tests followed the criteria recommended by a report of the Joint FAO/IAEA Expert Consultation [23]. The recovery results for the three laboratories are shown in Table 2. The R^2^ of the calibration curves for PVAc were >0.999 at all laboratories. The recovery ranges for the laboratories were 90.0–93.7%, 96.7–101.6%, and 100.4–114.2% at 300, 500, and 700 µg/mL, respectively. The RSD (%) range of the three laboratories was 1.7–5.3%. Briefly, the PVAc recoveries were in the range of 70–120% set by the Codex Alimentarius Commission, and the RSD (%) results were less than the 20% value defined in the EU Directive SANCO/2007/3131 [2,20].

### 3.4. Applying the Method to Gum Samples

The developed analytical method using HPLC–ELSD was used to determine the content of PVAc in commercial chewing gum purchased from domestic and internet markets. The concentrations of PVAc obtained from the individual sample analyses are shown in Table 3. A total of 20 chewing gum samples were analyzed, and PVAc detected in 17 samples originating from Korea, USA, Spain, Japan, Canada, and Germany. PVAc was not detected in 3 samples, labeled as natural chicle gum from Korea and Germany. The PVAc levels in the positive samples were 4.3–13.8 g/kg.

In order to confirm the presence of PVAc in the gum sample, it was analyzed by the method developed in this study, using Py–GC–MS. Pyrograms of the positive samples identified the typical pyrolysis products, such as acetic acid, benzene, toluene, styrene, indene, naphthalene, and acenaphthene (Figure 3b). However, in the negative samples, only small amounts of three components (acetic acid, toluene, and styrene) were detected, as mentioned above (see Figure 3c, Py–GC–MS analysis). The developed HPLC–ELSD and Py–GC–MS methods have shown good applicability in the PVAc analysis of chewing gum.

## 4. Conclusions

In this study, we have developed a method for quantitative and qualitative analysis of PVAc in chewing gum using HPLC–ELSD and Py–GC–MS. The developed HPLC–ELSD method showed good linearity, low LOD, and high accuracy and precision, meeting all of the criteria set by the Codex Alimentarius Commission and the EU Directive SANCO/2007/3131 (EC, 2007). This method was applied to determine the PVAc of commercially available chewing gum purchased from domestic and internet markets. In addition, this quantitative analytical method was verified by Py–GC–MS analysis, and found the typical pyrolysates of PVAc, such as acetic acid, benzene, toluene, styrene, indane, naphthalene, and acenaphthene. This method can be applied for the successful identification of PVAc in chewing gum products.

## Figures and Tables

**Table 1:** Results of accuracy and precision for PVAc using HPLC–ELSD.

Conc. (μg/mL)	Inter-day test (*n* = 3)		
	Measured concentration (μg/mL)	Accuracy (Recovery%)	Precision (RSD%)
200	186.3 ± 8.0	86.6 ± 5.6	6.4
300	310.3 ± 22.3	103.4 ± 7.4	7.2
400	408.1 ± 6.0	115.3 ± 3.9	3.4
500	521.0 ± 39.0	104.2 ± 7.8	7.5
600	622.8 ± 15.6	107.5 ± 3.7	3.5
700	776.6 ± 32.4	110.9 ± 4.6	4.2
800	799.8 ± 10.0	98.6 ± 0.6	0.6
900	900.2 ± 22.6	100.0 ± 2.5	2.5
1000	983.9 ± 4.8	93.8 ± 4.0	4.2
Conc. (μg/mL)	Intra-day test (*n* = 5)		
	Measured concentration (μg/mL)	Accuracy (Recovery%)	Precision (RSD%)
200	201.9 ± 6.3	101.0 ± 3.1	3.1
300	306.4 ± 25.1	100.8 ± 3.5	3.4
400	454.2 ± 16.5	113.6 ± 4.1	3.6
500	587.6 ± 33.4	105.2 ± 9.5	9.0
600	643.1 ± 17.1	107.2 ± 2.8	2.7
700	746.8 ± 23.8	115.0 ± 0.5	0.4
800	798.2 ± 507	99.8 ± 0.9	0.9
900	885.4 ± 25.8	101.0 ± 1.8	1.8
1000	980.7 ± 19.8	98.1 ± 2.0	2.0

RSD means relative standard deviation.

**Table 2:** Results of the cross-laboratory test for PVAc using HPLC–ELSD (*n* = 3).

		Lab 1		Lab 2		Lab 3		Precision (RSD%)
		Measured Concentration (μg/mL)	Accuracy (Recovery%)	Measured Concentration (μg/mL)	Accuracy (Recovery%)	Measured Concentration (μg/mL)	Accuracy (Recovery%)	
Concentration (μg/mL)	300	279.0 ± 11.3	93.0 ± 4.1	287.0 ± 3.5	93.7 ± 3.6	269.9 ± 2.1	90.0 ± 0.7	1.7
	500	508.0 ± 8.0	101.6 ± 1.6	483.3 ± 3.1	96.7 ± 0.6	503.7 ± 5.0	100.7 ± 1.0	2.1
	700	761.0 ± 16.5	108.7 ± 2.2	897.3 ± 5.6	114.2 ± 0.5	703.0 ± 9.3	100.4 ± 1.3	5.3
Linearity (R^2^)		0.9926		0.9965		0.9990		

**Table 3:** Concentration of PVAc in chewing gum samples.

Sample Information (Purchased Location)	Origin	PVAc in Gum (g/kg)
Gum 1 (Korea)	Cheongju, Korea	8.4 ± 0.1
Gum 2 (Korea)	Cheongju, Korea	10.8 ± 0.1
Gum 3 (Korea)	Cheongju, Korea	9.8 ± 0.0
Gum 4 (Korea)	Cheongju, Korea	9.0 ± 0.0
Gum 5 (Korea)	Cheongju, Korea	n.d. ^a^
Gum 6 (Korea)	Cheongju, Korea	9.1 ± 0.1
Gum 7 (Internet)	Madrid, Spain	5.1 ± 0.2
Gum 8 (Internet)	Madrid, Spain	4.9 ± 0.1
Gum 9 (Internet)	Madrid, Spain	5.7 ± 0.0
Gum 10 (Internet)	Chicago, USA	13.8 ± 0.1
Gum 11 (Internet)	Chicago, USA	4.3 ± 0.1
Gum 12 (Internet)	Chicago, USA	13.2 ± 0.3
Gum 13 (Internet)	Hanover, USA	6.3 ± 0.1
Gum 14 (Korea)	Japan	6.5 ± 0.0
Gum 15 (Korea)	Japan	6.9 ± 0.1
Gum 16 (Korea)	Japan	5.2 ± 0.0
Gum 17 (Korea)	Japan	6.9 ± 0.1
Gum 18 (Internet)	Langley, Canada	12.2 ± 0.1
Gum 19 (Internet)	Germany	n.d.
Gum 20 (Internet)	Germany	n.d.

^a^ n.d. indicates not detected.

## Supplementary Materials

The following are available online at https://www.mdpi.com/2304-8158/9/10/1473/s1, Table S1: TMR-GC-MS conditions applied in this study.

## References

[1] KoSFoST (Korean Society of Food Science and Technology) Polyvinyl Acetate, Food Science and Technology Dictionary. https://terms.naver.com/entry.nhn?docId=296974&cid=602-62&categoryId=60262.

[2] Assembly of Life Sciences (U.S.), Food and Nutrition Board, Committee on Codex Specifications (1981). Food Chemicals Codex Issue 1 of Food Chemicals Codex: First Supplement to the Third Edition, National Research Council (U.S.): Committee on Food Chemicals Codex.

[3] MFDS (Ministry of Food and Drug Safety) (2018). Korea Food Additives Code.

[4] JFCRF (The Japan Food Chemical Research Foundation) Standards for Use According to Use Categories Japan. https://www.ffcr.or.jp/en/upload/Standards%20for%20Use%20Jan.15.2020.pdf.

[5] FDA (U.S. Food and Drug Administration) Substances Added to Food (Formerly EAFUS). https://www.accessdata.fda.gov/scripts/fdcc/index.cfm?set=FoodSubstances&id=POLYVINYLACETATE.

[6] Spectrum Chemical MFG Corp Polyvinyl Acetate MSDS. https://www.spectrum-chemical.com/MSDS/ZQ446.pdf.

[7] IARC (International Agency for Research on Cancer) (1971). Vinyl acetate, polyvinyl acetate and polyvinyl alcohol. iarc monographs on the identification of the carcinogenic hazards to humans. IARC Monogr. Eval. Carcinog. Risk Chem. Hum..

[8] Sellier N., Jones C.E., Guiochon G. (1975). The examination of some vinyl acetate/olefin copolymers by pyrolysis gas chromatography mass spectrometry. J. Chromatogr. Sci..

[9] Tsuge S., Ohtani H., Watanabe C. (2011). Poly (vinyl acetate). PVAC: Pyrolysis-GC/MS Data Synthetic Polymers.

[10] Uyar T., Tonelli A.E., Hacaloglu J. (2006). Thermal degradation of polycarbonate, poly (vinyl acetate) and their blends. Polym. Degrad. Stab..

[11] Renuka Devi K.B., Madivanane R. (2012). Normal coordinate analysis of Polyvinyl acetate. Eng. Sci. Technol. Int. J..

[12] Bianco G., Zianni R., Anzillotta G., Palma A., Vitacco V., Scrano L., Cataldi T.R. (2013). Dibenzo-p-dioxins and dibenzofurans in human breast milk collected in the area of Taranto (Southern Italy): First case study. Anal. Bioanal. Chem..

[13] NIFDS (National Institutte of Food and Drug Safety Evaluation) (2015). Guidelines for the Validation of Drugs.

[14] KFDA (Korea Food and Drug Administration) (2013). Korean Food Additives Code, Standards and Specifications for Food Additives.

[15] EMEA (European Medicines Agency) (2006). Validation of Analytical Procedures: Text and Methodology.

[16] Ur-Rehman S., Mushtaq Z., Zahoor T., Jamil A., Murtaza M.A. (2015). Xylitol: A review on bioproduction, application, health benefits, and related safety issues. Crit. Rev. Food Sci. Nutr..

[17] Andrew F., Ajmol A., Nicholas G. (2009). Caffeine enhances cognitive function and skill performance during simulated soccer activity. Int. J. Sport Nutr. Exerc. Metab..

[18] Caprita A., Caprita R., Simulescu Gianet V.O., Drehe R.-M. (2010). Dietary fiber: Chemical and functional properties. J. Agroaliment. Process. Technol..

[19] (2015). The 80th JECFA Monographs 17. http://www.fao.org/fileadmin/user_upload/jec-fa_additives/docs/monograph17/additive-542-m17.pdf.

[20] Lee P.J., Yang J., Di Gioia A.J. (2013). Waters Polymer Analysis Applications Note.

[21] Chong H.S., Sim S., Yamaguchi T., Park J.H., Lee C., Kim M.K., Lee G.Y., Yun S.S., Lim H.S., Suh H.J. (2019). Simultaneous determination of sodium iron chlorophyllin and sodium copper chlorophyllin in food using high-performance liquid chromatography and ultra-performance liquid chromatography–mass spectrometry. Food Chem..

[22] EC (European Commission) (2007). Method Validation and Quality Control Procedures for Pesticide Residue Analysis in Food and Feed.

[23] FAO (Food and Agriculture Organization) (1998). Validation of Analytical Methods for Food Control.

