# Supplementary material for: Determination of Polyvinyl Acetate in Chewing Gum Using High-Performance Liquid Chromatography–Evaporative Light Scattering Detector and Pyrolyzer–Gas Chromatography–Mass Spectrometry
Source: Foods. 2020 Oct 15;9(10):1473. doi: 10.3390/foods9101473 (PMC7602758; doi:10.3390/foods9101473)
Supplement: Supplementary file 1 [file foods-09-01473-s001.pdf]

foods

Supplementary Materials:

Table S1. TMR-GC-MS conditions applied in this study.

Instrument		Condition
Py	Furnace	400 °C
	Interface	320 °C
GC	Inlet	320 °C, split ratio 200:1
	Oven	40 °C (3 min hold) → 20 °C/min → 320 °C (5 min hold)
	Column	UA-5<br>(30 m length × 0.25 mm inner diameter × 0.25 µm film thickness)
MS		Scan range, m/z 10~550<br>Scan speed, 5.19 scans/sec
